# Supplementary material for: An inducible tricolor reporter mouse for simultaneous imaging of lysosomes, mitochondria and microtubules
Source: bioRxiv. 2023 May 22:2023.05.22.541817. Preprint. [Version 1] doi: 10.1101/2023.05.22.541817 (PMC10245888; doi:10.1101/2023.05.22.541817)
Supplement: Supplement 8 [file NIHPP2023.05.22.541817v1-supplement-8.pdf]

bioRxiv preprint doi: https://doi.org/10.1101/2023.05.22.541817; this version posted May 23, 2023. The copyright holder for this preprint (which was not certified by peer review) is the author/funder, who has granted bioRxiv a license to display the preprint in perpetuity. It is made available under aCC-BY 4.0 International license.

Supplementary Figures

SupFig. 1: Additional details on the design and validation of the Kaleidoscope reporter.

(**A**) Intermediate vector to show cloning sites to concatenate the 3 fusion proteins. The bolded MluI sites used to transfer them to the Ai9 targeting vector to generate the ROSA-Kaleidoscope

bioRxiv preprint doi: https://doi.org/10.1101/2023.05.22.541817; this version posted May 23, 2023. The copyright holder for this preprint (which was not certified by peer review) is the author/funder, who has granted bioRxiv a license to display the preprint in perpetuity. It is made available under aCC-BY 4.0 International license.

plasmid. The Lamp1 sequence is from rat, while the MLS and alpha-Tubulin sequences are from human. See Table 1 for cloning primer sequences and the order of cloning. (**B**) MLE15 cells transfected with Cre-recombined ROSA-Kaleidoscope to show comparable fluorescence patterns as in HEK 293T cells. Scale: 10 um. (**C**) Expected PCR genotyping results of $ROSA^{Kaleidoscope}$ littermates. See Table 2 for genotyping primer sequences. (**D**) Western blots of lungs expressing all 3 fusion proteins and HEK 293T cells expressing individual fusion proteins. The second largest band specific to the Kaleidoscope lungs might be due to incomplete glycosylation of LAMP1. Both BFP and mKate2 are tagRFP derivatives and detected by the same antibody.

SupFig. 2: Additional characterization of the Kaleidoscope reporter in organs.

(**A**) A single optical section of the skeleton muscle in Fig. 2B to show that microtubules are aligned perpendicular to actomyosin bundles, whereas mitochondria are along and between actomyosin bundles (double arrow). s, satellite cell. Scale: 10 um. (**B**) A low magnification view of the liver showing inefficient recombination by CMV-Cre. Boxed area is shown in Fig. 2B. Scale: 50 um. (**C**) Distribution of Kaleidoscope fusion proteins in airway ciliated cells with characteristic apical microtubule clusters. Tam, 500 ug tamoxifen. Scale: 10 um. (**D**) Distribution of Kaleidoscope fusion proteins in dome-shaped airway club cells and spindle-shaped airway smooth muscle cells. Scale: 10 um.

SupFig. 3: Generation of $Car4^{CreER}$ mice and high-magnification views of microtubules in lung cells.

(**A**) CRISPR targeting of the *Car4* locus. The gRNA sequence is shown with the protospacer adjacent motif (PAM; not included in gRNA) underlined; a cryptic translation start codon of *Car4* (ATG in red) is replaced by that of CreER. Locus-specific PCR genotyping identifies two positive mice. The primer sequences are: #1, 5'-TGTACTGCTATTCCTTGTTCATCT; #2, 5'-AGAAGCATTTTCCAGGTATG; #3, 5'-TGACACTGAGAACCACAAACGGC; #4, 5'-GTTCGCAAGAACCTGATGGACA; #5, 5'-TTCTAGTTGTGGTTTGTCCAAACT; #6, 5'-AACGTGAACAGCTACAAGGCACTG. (**B**) $Car4^{CreER}$ targets Cap2 endothelial cells expressing ERG and CAR4 with characteristic large net-like morphology. Further characterization will be described in another study. Scale: 10 um. (**C**) Filamentous microtubules are apparent in thin z stacks (<5 um) of labelled AT2, AT1, and Cap2 cells (arrowhead and bracket). n, nucleus. See Fig. 4 for experimental conditions. Scale: 10 um.

SupFig. 4: Additional examples of labelled AT1 cells upon Sendai virus infection.

Low-magnification view of individual labeled AT1 cells in uninfected lungs and unaffected and AT2-less regions (devoid of LAMP3+ AT2 cells). Boxed regions are magnified. Mitochondrial fluorescence of the AT1 cell in the AT2-less region is not as reduced as in Fig. 5 possibly reflecting an earlier stage of cell displacement. Scale: 10 um.

SupFig. 5: Further characterization of labeled epithelial cells in the *Yap/Taz* mutant

bioRxiv preprint doi: https://doi.org/10.1101/2023.05.22.541817; this version posted May 23, 2023. The copyright holder for this preprint (which was not certified by peer review) is the author/funder, who has granted bioRxiv a license to display the preprint in perpetuity. It is made available under aCC-BY 4.0 International license.

(**A**) Labeled LAMP3- airway cells still contain BFP-Lamp1 puncta. Tam, 500 ug tamoxifen. Scale: 10 um. (**B**) En face view and perimeter quantification of alveolar epithelial cells outlined with E-Cadherin (ECAD) immunostaining. Cells in the *Yap/Taz* mutant have an intermediate perimeter between AT1 and AT2 cells of the control lung. Tam, 3 mg tamoxifen. Asterisk, $p<0.0001$ (ordinary ANOVA with Tukey test; data are mean and standard deviation). Scale: 10 um.

SupVideo 1: 3D view of labeled AT1 cells, as in Fig. 4A.

SupVideo 2: 3D view of labeled Cap2 cells, as in Fig. 4A.

SupVideo 3: A 4-hr movie (4 min/frame) of live imaging of Sox9^{CreER} labeled epithelial cells in explanted embryonic lungs, as in Fig. 5A, showing all 3 fusion proteins.

SupVideo 4: A 4-hr movie (4 min/frame) of live imaging of Sox9^{CreER} labeled epithelial cells in explanted embryonic lungs, as in Fig. 5A, showing BFP-Lamp1.

SupVideo 5: A 4-hr movie (4 min/frame) of live imaging of Sox9^{CreER} labeled epithelial cells in explanted embryonic lungs, as in Fig. 5A, showing MLS-mKate2.

SupVideo 6: A 4-hr movie (4 min/frame) of live imaging of Sox9^{CreER} labeled epithelial cells in explanted embryonic lungs, as in Fig. 5A, showing EGFP-alpha-Tubulin.

SupVideo 7: 3D view of *Sftpc*^{CreER} lineage-labeled AT1 and AT2 cells, as in Fig. 6B.

bioRxiv preprint doi: https://doi.org/10.1101/2023.05.22.541817; this version posted May 23, 2023. The copyright holder for this preprint (which was not certified by peer review) is the author/funder, who has granted bioRxiv a license to display the preprint in perpetuity. It is made available under aCC-BY 4.0 International license.

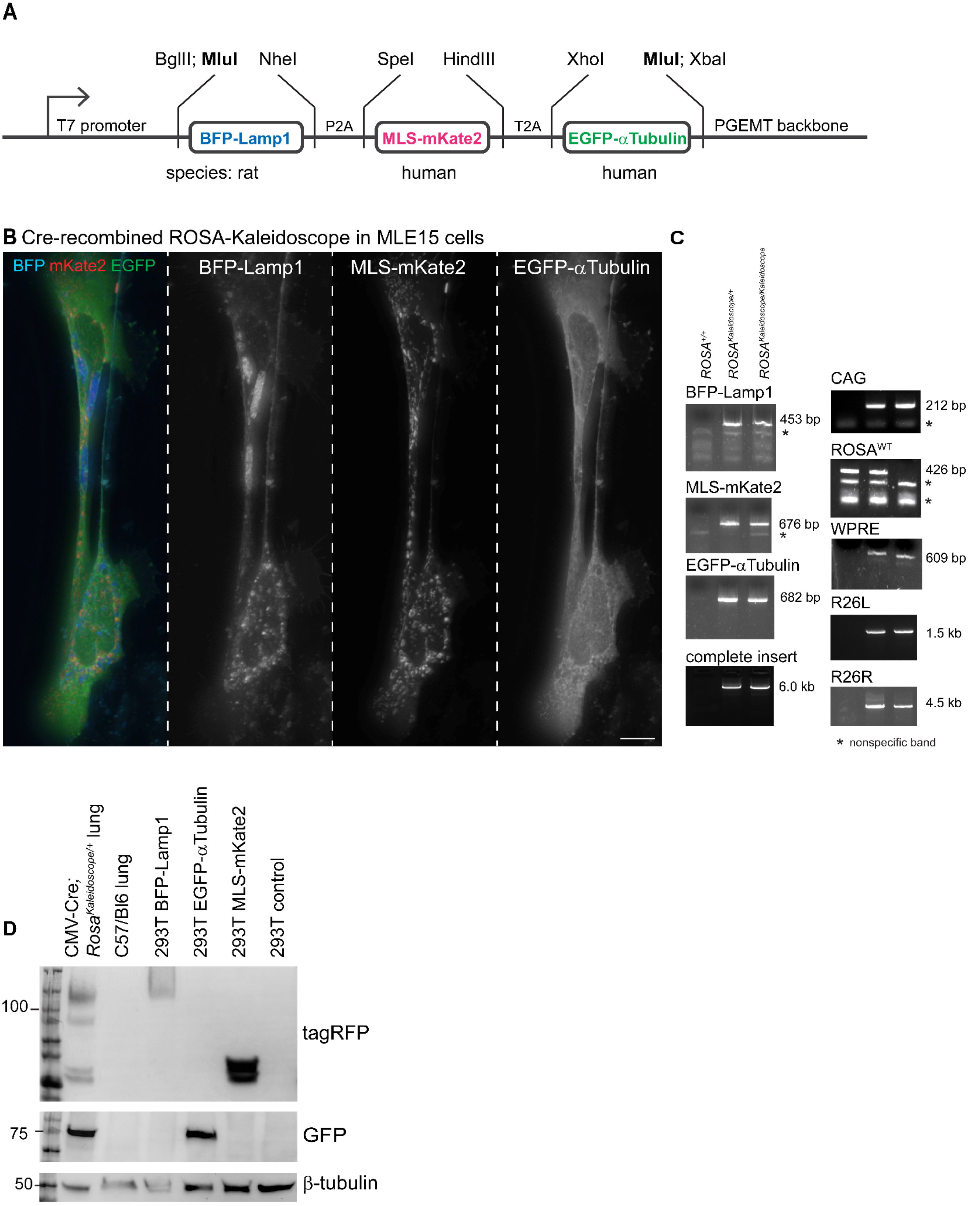

bioRxiv preprint doi: https://doi.org/10.1101/2023.05.22.541817; this version posted May 23, 2023. The copyright holder for this preprint (which was not certified by peer review) is the author/funder, who has granted bioRxiv a license to display the preprint in perpetuity. It is made available under aCC-BY 4.0 International license.

SupFig. 1: Additional details on the design and validation of the Kaleidoscope reporter.

(A) Intermediate vector to show cloning sites to concatenate the 3 fusion proteins. The bolded MluI sites used to transfer them to the Ai9 targeting vector to generate the ROSA-Kaleidoscope plasmid. The Lamp1 sequence is from rat, while the MLS and alpha-Tubulin sequences are from human. See Table 1 for cloning primer sequences and the order of cloning. (**B**) MLE15 cells transfected with Cre-recombined ROSA-Kaleidoscope to show comparable fluorescence patterns as in HEK 293T cells. Scale: 10 um. (**C**) Expected PCR genotyping results of $ROSA^{Kaleidoscope}$ littermates. See Table 2 for genotyping primer sequences. (**D**) Western blots of lungs expressing all 3 fusion proteins and HEK 293T cells expressing individual fusion proteins. The second largest band specific to the Kaleidoscope lungs might be due to incomplete glycosylation of LAMP1. Both BFP and mKate2 are tagRFP derivatives and detected by the same antibody.

bioRxiv preprint doi: https://doi.org/10.1101/2023.05.22.541817; this version posted May 23, 2023. The copyright holder for this preprint (which was not certified by peer review) is the author/funder, who has granted bioRxiv a license to display the preprint in perpetuity. It is made available under aCC-BY 4.0 International license.

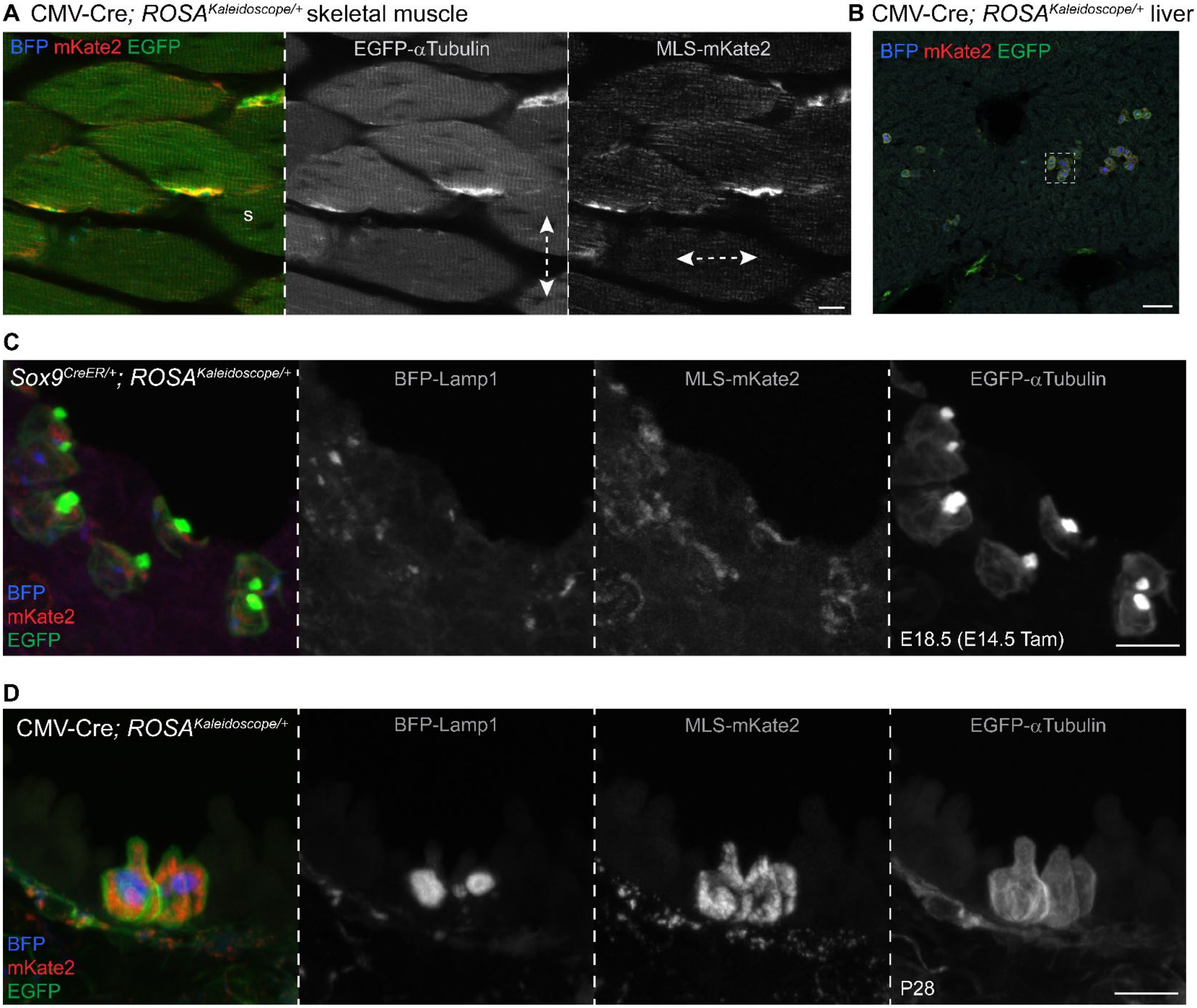

SupFig. 2: Additional characterization of the Kaleidoscope reporter in organs.

(**A**) A single optical section of the skeleton muscle in Fig. 2B to show that microtubules are aligned perpendicular to actomyosin bundles, whereas mitochondria are along and between actomyosin bundles (double arrow). s, satellite cell. Scale: 10 um. (**B**) A low magnification view of the liver showing inefficient recombination by CMV-Cre. Boxed area is shown in Fig. 2B. Scale: 50 um. (**C**) Distribution of Kaleidoscope fusion proteins in airway ciliated cells with characteristic apical microtubule clusters. Tam, 500 ug tamoxifen. Scale: 10 um. (**D**) Distribution of Kaleidoscope fusion proteins in dome-shaped airway club cells and spindle-shaped airway smooth muscle cells. Scale: 10 um.

bioRxiv preprint doi: https://doi.org/10.1101/2023.05.22.541817; this version posted May 23, 2023. The copyright holder for this preprint (which was not certified by peer review) is the author/funder, who has granted bioRxiv a license to display the preprint in perpetuity. It is made available under aCC-BY 4.0 International license.

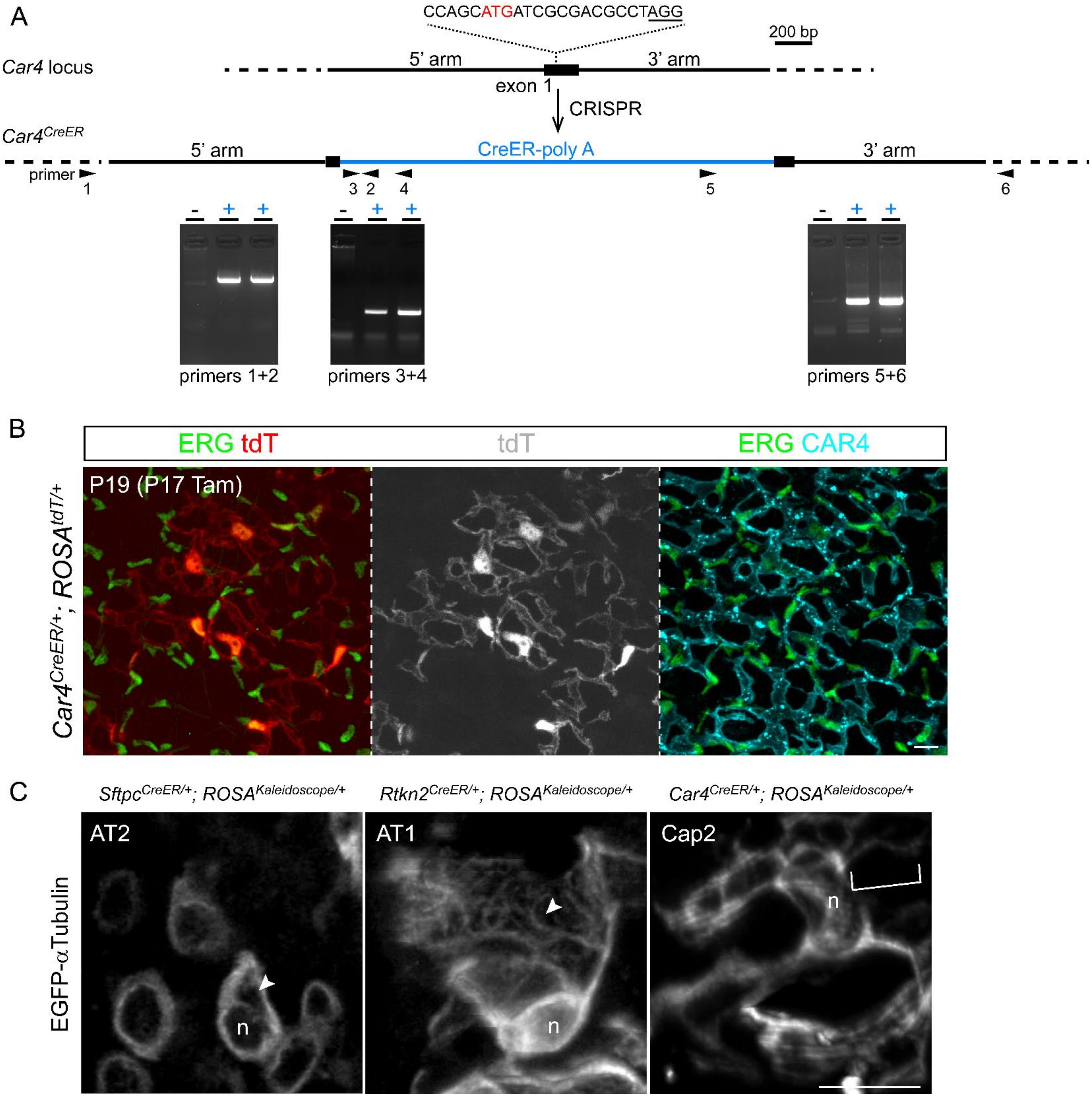

SupFig. 3: Generation of *Car4*^{CreER} mice and high-magnification views of microtubules in lung cells.

(**A**) CRISPR targeting of the *Car4* locus. The gRNA sequence is shown with the protospacer adjacent motif (PAM; not included in gRNA) underlined; a cryptic translation start codon of *Car4* (ATG in red) is replaced by that of CreER. Locus-specific PCR genotyping identifies two positive mice. The primer sequences are: #1, 5'-TGTACTGCTATTCCTTGTTCATCT; #2, 5'-AGAAGCATTTTCCAGGTATG; #3, 5'-TGACACTGAGAACCACAAACGGC; #4, 5'-GTTCGCAAGAACCTGATGGACA; #5, 5'-TTCTAGTTGTGGTTTGTCCAAACT; #6, 5'-AACGTGAACAGCTACAAGGCACTG. (**B**) *Car4*^{CreER} targets Cap2 endothelial cells expressing ERG and CAR4 with characteristic large net-like morphology. Further characterization will be described in another study. Scale: 10 um. (**C**) Filamentous microtubules are apparent in thin z stacks (<5 um) of labelled AT2, AT1, and Cap2 cells (arrowhead and bracket). n, nucleus. See Fig. 4 for experimental conditions. Scale: 10 um.

bioRxiv preprint doi: https://doi.org/10.1101/2023.05.22.541817; this version posted May 23, 2023. The copyright holder for this preprint (which was not certified by peer review) is the author/funder, who has granted bioRxiv a license to display the preprint in perpetuity. It is made available under aCC-BY 4.0 International license.

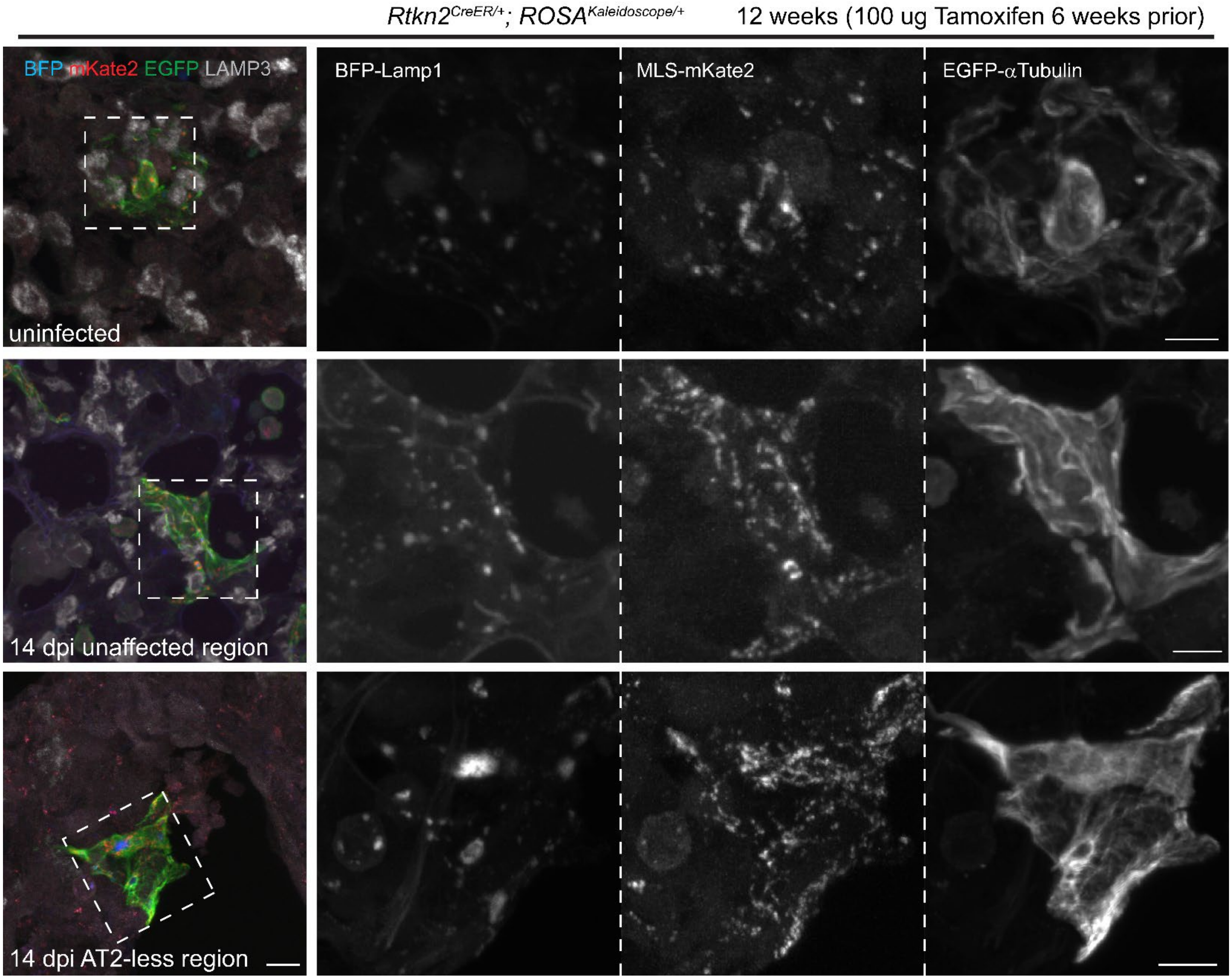

SupFig. 4: Additional examples of labelled AT1 cells upon Sendai virus infection.

Low-magnification view of individual labeled AT1 cells in uninfected lungs and unaffected and AT2-less regions (devoid of LAMP3+ AT2 cells). Boxed regions are magnified. Mitochondrial fluorescence of the AT1 cell in the AT2-less region is not as reduced as in Fig. 5 possibly reflecting an earlier stage of cell displacement. Scale: 10 um.

bioRxiv preprint doi: https://doi.org/10.1101/2023.05.22.541817; this version posted May 23, 2023. The copyright holder for this preprint (which was not certified by peer review) is the author/funder, who has granted bioRxiv a license to display the preprint in perpetuity. It is made available under aCC-BY 4.0 International license.

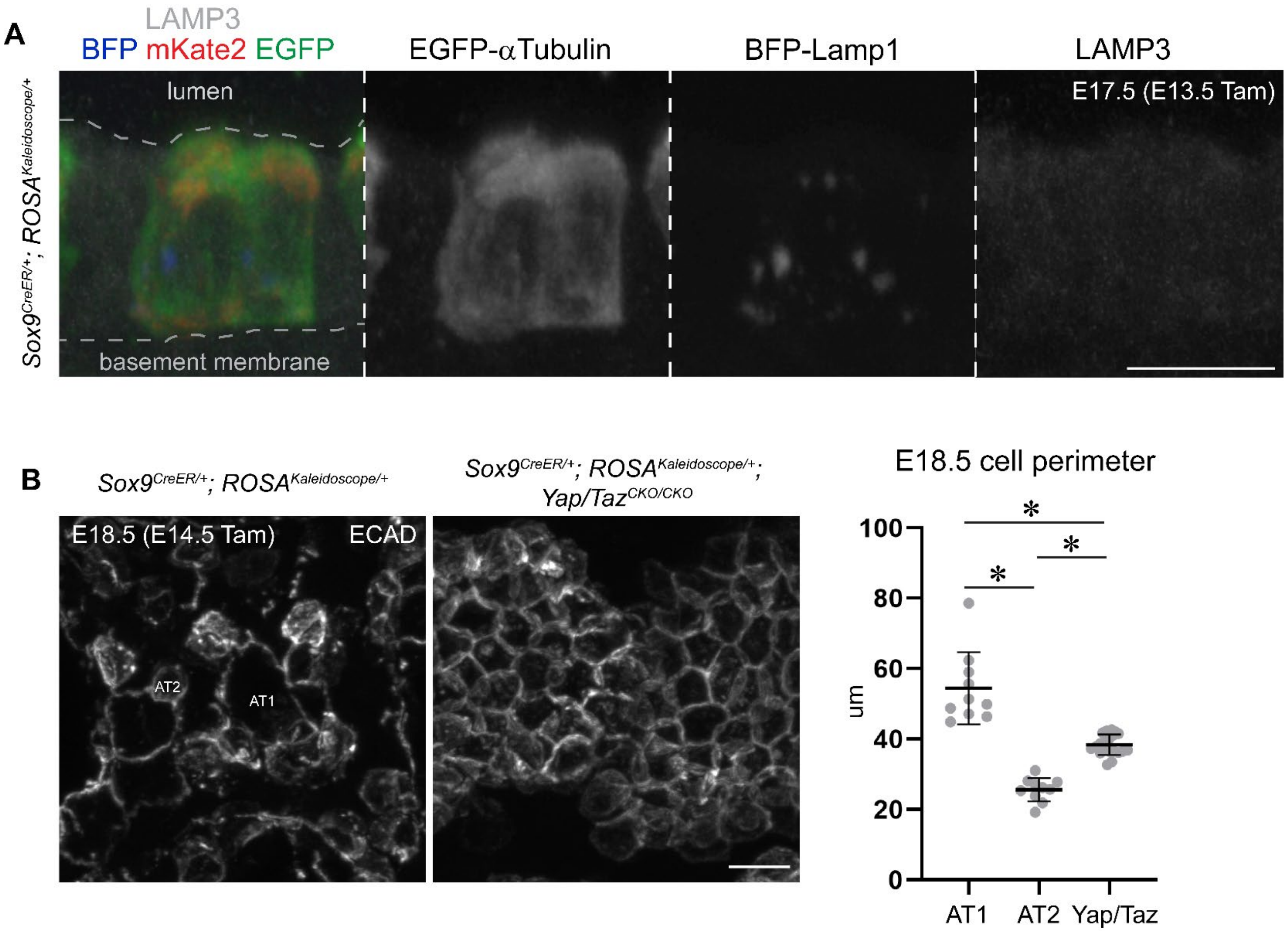

SupFig. 5: Further characterization of labeled epithelial cells in the *Yap/Taz* mutant

(**A**) Labeled LAMP3- airway cells still contain BFP-Lamp1 puncta. Tam, 500 ug tamoxifen. Scale: 10 um. (**B**) En face view and perimeter quantification of alveolar epithelial cells outlined with E-Cadherin (ECAD) immunostaining. Cells in the *Yap/Taz* mutant have an intermediate perimeter between AT1 and AT2 cells of the control lung. Tam, 3 mg tamoxifen. Asterisk, $p<0.0001$ (ordinary ANOVA with Tukey test; data are mean and standard deviation). Scale: 10 um.
